# The effect of improving psychosocial stressors on psychological distress: a quasi-experiment of Finnish health and social care workers

**DOI:** 10.1177/14034948241242160

**Published:** 2024-04-03

**Authors:** Risto Nikunlaakso, Rahman Shiri, Tuula Oksanen, Jaana Laitinen

**Affiliations:** 1 Finnish Institute of Occupational Health, Helsinki, Uusimaa, Finland; 2 University of Eastern Finland, Kuopio, Pohjois-Savo, Finland

**Keywords:** Work stress, mental health, interventions

## Abstract

**Aims:**

To examine how a positive change in one to three psychosocial stressors (job demands, job rewards, and workplace social capital) influenced psychological distress.

**Methods:**

The analysis included 3605 Finnish health and social services workers who completed surveys in 2019, 2020 and 2021. A logistic regression model was used to estimate the propensity score of experiencing a positive change in one to three psychosocial stressors between 2019 and 2020. We balanced the baseline characteristics with propensity scoring. A generalised linear model with a binomial distribution and a log link function was used to compare the quasi-intervention and quasi-control groups for the risk of psychological distress in 2021.

**Results:**

Among the total sample, neither improving a single stressor nor two or three stressors affected psychological distress. However, among employees younger than 50 years, improving two or three psychosocial stressors in 2019–2020 decreased the risk of moderate to severe psychological distress in 2021 by 41% (risk ratio 0.59, 95% confidence interval 0.36–0.96). Among employees aged 50 years or older, improving job rewards lowered the risk of mild to severe psychological distress by 23% (risk ratio 0.77, 95% confidence interval 0.62–0.96).

**Conclusions:**

**The results of this quasi-experiment suggest that the positive effect of improving psychosocial stressors is stronger among younger than older workers. Future interventions should be customised for different ages and aim to improve accumulated work stressors and individual stress-coping skills.**

## Background

Health and social services workers face many psychosocial work stressors at their workplace [1]. The COVID-19 pandemic has exacerbated the situation by increasing job demands and decreasing job rewards for many workers [2]. Simultaneously, the prevalence of mental health problems such as psychological distress and burnout has increased [3]. Psychosocial work stressors can increase the risk of developing mental health problems [4]. Moreover, psychological distress, for example, can lead to sickness absences and early retirement, which potentially further increases workload for the remaining workers [5].

Health and social service organisations urgently need effective interventions to reduce mental health problems. Most mental health interventions aim to help workers to cope with psychosocial stressors rather than address the stressors themselves [6]. Organisational-level interventions, aiming to improve working conditions, could produce more sustainable effects than interventions for individual workers [7]. They are, however, considerably less often studied [6]. Organisational-level interventions are, on the other hand, challenging to implement, and their effectiveness in reducing psychosocial work stressors is not well established [6, 7]. More research on reducing psychosocial work stressors is needed to support future organisational-level mental health interventions.

Psychosocial work stressors may affect employee health when acting independently and especially when acting jointly. The accumulation of several work stressors can increase the risk for low work ability and early retirement due to mental health reasons [8, 9]. Moreover, the accumulation can lead to synergistic interaction between stressors, which may have a stronger effect on mental health than the effect of the stressors acting separately [10]. According to Tsai and Venkataramani [11], when adverse health conditions accumulate and synergistically interact, an intervention that addresses all exposures jointly is more efficient than an intervention addressing only one factor. We previously [10] showed that high job demands, low job rewards, and low workplace social capital can synergistically interact and increase the risk for psychological distress. The study suggested that interventions should address all interacting stressors jointly. However, the effect of reducing accumulated work stressors on mental health symptoms remains unexamined. This could enable designing mental health interventions that tackle accumulated work stressors.

Quasi-experiments are potential alternatives for intervention studies, as they can enable causal inference and are often more feasible to conduct than randomised controlled trials (RCTs) [12]. They can be useful, for example, to test the feasibility of organisational-level interventions, which are often difficult to implement [12] and thus are considerably less often studied [6]. A few quasi-experiments have been conducted on Occupational health outcomes [13–15]. They have found fairness of managerial behaviour [13] and elimination of job strain [14] to reduce insomnia symptoms. Moreover, using participatory working time scheduling software was found to reduce the risk of employees’ low control over the scheduling of shifts, short sleep and poor workability [15]. However, to our knowledge, no quasi-experiments have been conducted on accumulated work stressors.

The aim of this quasi-experimental study was to investigate whether improving psychosocial work stressors decreases the risk of psychological distress among health and social services workers. We investigated the effect of improving job demands, job rewards and workplace social capital, which are common factors influencing the working environment in social and healthcare workplaces and the main exposures for mental health-related sickness absences [4]. As the prevalence of psychosocial work stressors and mental health problems can vary in different ages [16, 17], we also conducted an age-specific analysis of improving work stressors.

## Methods

### Participants

The study baseline sample included 12,687 employees from four Finnish public health and social care districts located in southern and eastern Finland who participated in an employee survey in 2019. The eligible population did not include workers who were on leave for parental, medical, or educational reasons. Of the participants, 9608 (76%) gave their written consent to use their survey responses for research purposes. Out of the 9608 baseline respondents, 6392 completed the first follow-up survey in 2020 and 4502 completed the second follow-up survey in 2021. Response rates were 68% in 2019, 66% in 2020 and 63% in 2021. After excluding participants with incomplete data, 3605 were included in the final sample. Appendix 1 shows the baseline characteristics of the eligible population, respondents of the baseline survey, and final sample. No differences were found between the populations regarding age or sex; minor differences were found regarding occupational groups. The ethical committee of the Finnish Institute of Occupational Health approved the study. Participation in the survey was voluntary and consent to use the answers for scientific research was asked for in the survey questionnaire.

### Variables

#### Outcome

The outcome of the study, psychological distress, was measured using the four-item patient health questionnaire (PHQ-4). Psychological distress at baseline in 2019 was measured with the general health questionnaire-12, but the instrument was changed to the PHQ-4 in the 2021 survey. The PHQ-4 comprises two items from the nine-item patient health questionnaire and seven-item generalised anxiety disorder, and is a valid screening instrument for psychological distress in the general population [18]. The items included, for example, ‘over the last 2 weeks, how often have you been bothered by the following problems: feeling nervous, anxious or on edge’. Responses were scored as 0 (‘not at all’), 1 (‘several days’), 2 (‘more than half the days’), or 3 (‘nearly every day’). The total score ranges between 0 and 12. Following the recommendation of Kroenke et al. [19], mild to severe psychological distress was defined as a PHQ-4 score of 3 or higher, and moderate to severe psychological distress was defined as a PHQ-4 score of 6 or higher.

#### Psychosocial stressors

In the survey used, job demands were measured with two items (baseline Cronbach’s alpha = 0.88): ‘An unreasonable amount of work is expected of me’ and ‘I don’t have enough time to get my work done’, with a five-point response scale (1 = strongly agree to 5 = strongly disagree). Items were derived from the job content questionnaire [20], which is validated among social and healthcare workers [21]. Job rewards were measured with three items (baseline Cronbach’s alpha = 0.71): ‘How much do you feel you get in return for work in terms of income and job benefits?’, ‘How much do you feel you get in return for work in terms of recognition and prestige?’, and ‘How much do you feel you get in return for work in terms of personal satisfaction?’, with a five-point response scale (1 = very much to 5 = not at all). Items were derived from the effort–reward imbalance questionnaire [22], validated among social and healthcare workers [23]. Workplace social capital was measured with eight items (baseline Cronbach’s alpha = 0.87) developed and validated among public sector workers by Kouvonen et al. [24]; for example, ‘We have a “we are together” attitude’, ‘People feel understood and accepted by each other’, and ‘Our supervisor treats us with kindness and consideration’. The response scale had five points (1 = strongly disagree to 5 = strongly agree in seven items, and 1 = to a very little extent to 5 = to a very great extent in one item). We calculated a mean of the scores for all psychosocial stressors.

#### Covariates

Age (continuous variable), sex, type of work contract (full-time vs. part-time), and occupation were collected from organisational registers. We classified the respondents into three levels of socioeconomic status according to their occupation: Skilled labour included for example managers, physiotherapists, physicians and nurses. Semi-skilled labour included for example practical nurses, social workers and clerical workers. Unskilled labour included for example cleaning and kitchen staff. Self-reported information on marital status (unmarried, married, registered relationship, cohabitation, divorced/separated or widowed), having a child (yes/no), shift work (regular daywork, shift work without night shifts, shift work with night shifts, regular night work, other irregular work), number of years of shift work, number of weekly working hours, and working as a supervisor (yes/no) were gathered in all surveys.

### Statistical analysis

We used logistic regression to calculate the propensity score of the conditional probability of experiencing a positive change in psychosocial work factors. A positive change (hereafter quasi-intervention) referred to one of these five scenarios from 2019 to 2020: (a) a decrease of at least one standard deviation (SD) in job demands; (b) an increase of at least one SD in job rewards; (c) an increase of at least one SD in workplace social capital; (d) a decrease of at least one SD in any of the three psychosocial factors; or (e) a decrease of at least one SD in two of the three psychosocial factors.

We included background characteristics and outcome variables measured in 2019 and 2020 in the propensity scores. The characteristics were: age (continuous), sex, socioeconomic status, marital status, having a child, type of work contract (full time or part time), shift work, number of years of shift work, number of weekly working hours, working as a supervisor, job demands (continuous), job rewards (continuous), workplace social capital (continuous), perceived general health (five categories), insomnia (measured with the Jenkins sleep scale, continuous), and psychological distress (measured with the general health questionnaire-12, continuous).

We computed an inverse probability of treatment weight for each participant using the propensity score. The weights were based on the inverse of their chance of experiencing a positive change in a psychosocial work factor between 2019 and 2020. We stabilised the inverse probability of treatment weights to reduce the variability and bias [25]. We also calculated age-specific propensity scores and inverse probability of treatment weights for employees aged less than 50 years and those aged 50 years and over, thus creating two groups of roughly similar size. To compare the quasi-intervention and quasi-control groups in terms of the risk of psychological distress in 2021, we applied a generalised linear model with a binomial distribution and a log link function. We used analytical weights for the inverse probability of treatment weight in the model.

We evaluated weighting effectiveness by checking the balance in baseline characteristics of the quasi-intervention and quasi-control group [26]. We estimated the unweighted and weighted prevalence and mean values of the baseline characteristics to compare the intervention and control groups. We also performed three kinds of sensitivity analyses among the total sample: (a) removing employees with moderate or severe psychological distress in 2019 or 2020 from the propensity scores; (b) including only employees with high job demands, low job rewards, or low workplace social capital in 2019 in the propensity scores; and (c) for the employees who had a significant decrease (at least one SD) in two out of the three psychosocial factors from 2019 to 2020, the comparison group consisted of employees who did not experience any positive change in their job demands, job rewards, or workplace social support during the same period. We used Stata, version 17 for the analyses.

## Results

Of the employees with no missing data on all the characteristics in 2019 and 2020 (*N*=3605), 88.6% were women and 41.5% were aged 50 years or older (Table I). Over 92% worked full time, 67% were skilled workers and 77.3% rated their general health good or fairly good.

**Table I.** Comparison of baseline characteristics in 2019 according to decrease in one of the three stressors between 2019 and 2020 (no vs. yes) in the unweighted and weighted samples, proportions (%) and means ± standard deviation (SD).

Characteristic	Unweighted		Weighted	
	No (*N*=2148) %	Yes (*N*=1457) %	No (*N*=2148) %	Yes (*N*=1457) %
Female sex	88.5	88.7	88.6	88.6
Age ⩾50 years	42.0	40.8	41.4	40.9
Marital status				
Unmarried	10.7	10.5	10.8	10.9
Married	57.3	54.4	56.2	56.7
Cohabitating	20.1	22.9	21.2	20.7
Divorced, separated, or widowed	11.9	12.2	11.8	11.7
Socioeconomic status				
Unskilled	3.8	3.0	3.6	3.8
Semi-skilled	27.6	32.3	29.7	29.3
Skilled	68.6	64.7	66.7	66.9
Type of work contract				
Full time	91.9	92.6	92.3	92.3
Part time	8.1	7.4	7.7	7.7
Shift workers	45.8	50.2	47.6	47.1
Having children	47.1	48.7	47.6	47.6
Working as a supervisor	7.2	7.0	7.0	7.3
Perceived general health				
Good	42.6	36.7	40.3	40.1
Fairly good	37.0	37.2	37.2	37.1
Average	17.5	21.8	19.2	19.4
Fairly poor or poor	2.9	4.3	3.3	3.4
Years of shiftwork				
0	30.0	26.7	29.2	27.9
1–10	36.2	36.2	36.4	36.0
>10	33.7	37.1	34.4	36.0
Weekly working hours				
<38	22.5	23.4	22.8	22.7
38–40	60.2	56.6	60.2	57.5
>40	17.3	20.1	17.1	19.7
Having sleep disturbances (JSS score >11)	18.7	23.0	20.4	19.4
Psychologically distressed (GHQ-12 score >2)	34.4	45.0	37.9	38.6
	Mean ± SD	Mean ± SD	Mean ± SD	Mean ± SD
Age (years)	45.8 ± 10.4	45.4 ± 10.4	45.6 ± 10.4	45.6 ± 10.3
Years of shiftwork	8.9 ± 9.8	9.3 ± 9.5	9.0 ± 9.8	9.0 ± 9.4
Weekly working hours	36.2 ± 10.3	36.0 ± 11.2	36.1 ± 10.5	36.1 ± 10.9
Insomnia	7.0 ± 4.7	7.7 ± 5.0	7.3 ± 4.8	7.3 ± 4.8
GHQ-12	2.48 ± 3.2	3.32 ± 3.7	2.79 ± 3.4	2.77 ± 3.4

JSS: Jenkins sleep scale; GHQ-12: general health questionnaire-12.

Between 2019 and 2020, job demands reduced one SD or more for 21.7% of the employees, and job rewards and workplace social capital improved for 18.3% and 14.8% of the employees, respectively (Table II). Only improvement in workplace social capital was marginally higher in employees younger than 50 years than in those aged 50 years or older (*P*=0.083). The weighting well balanced the measured characteristics between the intervention and control groups (Table I), and there were no statistically significant differences in 2019 or 2020 characteristics between the two groups.

**Table II.** Proportion (%) of employees with change in psychosocial stressors between 2019 and 2020.

Psychosocial stressor	<50 years	>50 years	All
Reduction of job demands	21.1	22.5	21.7
Improvement in job rewards	18.8	17.6	18.3
Improvement in workplace social capital	15.6	13.5	14.8
Improvement of one of the three stressors	40.9	39.7	40.4
Improvement of at least two of the three stressors	11.4	11.6	11.5

The prevalence of mild to severe psychological distress was 36.4%, being higher in employees younger than 50 years than in those aged 50 years or older (39.0% vs. 32.8%, *P*<0.001). The prevalence of moderate or severe psychological distress was 9.5%. It was also significantly higher in employees aged less than 50 years than in those aged 50 years or older (10.8% vs. 7.8%, *P*=0.003).

Improvement in job rewards between 2019 and 2020 had a small and statistically insignificant effect on the risk of mild to severe psychosocial distress in 2021 (risk ratio (RR) 0.90, 95% confidence interval (CI) 0.80–1.02, *P*=0.092, Table III) but had no effect on the risk of moderate or severe psychosocial distress. Reduction in job demands, improvement in workplace social capital, and improving at least one or two of the three work stressors did not reduce the risk of psychological distress.

**Table III.** The effects of psychosocial risk improvement between 2019 and 2020 on psychological distress in 2021.

Psychosocial stressor	*N*	Mild to severe distress			Moderate to severe distress		
		%	RR	95% CI	%	RR	95% CI
Reduction in job demands							
No	2785	36.7	1		9.6	1	
Yes	748	36.3	0.99	0.89–1.10	8.9	0.92	0.71–1.19
Improvement in job rewards							
No	2895	37.1	1		9.3	1	
Yes	638	33.5	0.90	0.80–1.02	9.7	1.04	0.80–1.36
Improvement in workplace social capital							
No	3029	36.4	1		9.4	1	
Yes	514	34.5	0.95	0.83–1.08	8.7	0.93	0.69–1.25
Improvement in at least one of the three stressors							
No	2130	37.3	1		9.6	1	
Yes	1446	35.3	0.95	0.87–1.03	9.5	0.99	0.80–1.21
Improvement in at least two of the three stressors							
No	3126	36.9	1		9.6	1	
Yes	407	34.5	0.94	0.81–1.08	8.0	0.84	0.59–1.19

CI: confidence interval; RR: risk ratio.

In age-specific analyses (Table IV), improvements in at least two of the three psychosocial stressors between 2019 and 2020 reduced the risk of moderate or severe psychological distress by 41% (RR 0.59, 95% CI 0.36–0.96) among employees younger than 50 years, and improvement in job rewards reduced the risk of mild to severe psychological distress by 23% (RR 0.77, 95% CI 0.62–0.96) among employees aged 50 years or older.

**Table IV.** The effects of psychosocial risk improvement between 2019 and 2020 on psychological distress in 2021 among workers younger than 50 years and those aged 50 years or older.

Psychosocial stressor	*N*	Mild to severe distress			Moderate to severe distress		
		%	RR	95% CI	%	RR	95% CI
Workers aged <50 years							
Reduction in job demands							
No	1636	39.6	1		11.2	1	
Yes	425	37.7	0.95	0.83–1.09	8.4	0.75	0.53–1.05
Improvement in job rewards							
No	1683	39.7	1		10.8	1	
Yes	384	37.9	0.95	0.83–1.10	11.6	1.08	0.79–1.47
Improvement in workplace social capital							
No	1743	39.1	1		10.9	1	
Yes	318	36.2	0.92	0.79–1.08	8.8	0.81	0.56–1.18
Improvement in at least one of the three stressors							
No	1237	40.3	1		11.5	1	
Yes	859	37.4	0.93	0.83–1.04	9.9	0.86	0.66–1.10
Improvement in at least two of the three stressors							
No	1823	39.9	1		11.1	1	
Yes	238	34.8	0.87	0.73–1.05	6.6	0.59	0.36–0.97
Workers aged ⩾50 years							
Reduction in job demands							
No	1143	32.4	1		7.2	1	
Yes	323	33.2	1.02	0.86–1.22	8.9	1.23	0.82–1.85
Improvement in job rewards							
No	1212	33.6	1		7.3	1	
Yes	254	25.9	0.77	0.62–0.96	7.1	0.97	0.60–1.59
Improvement in workplace social capital							
No	1270	32.5	1		7.2	1	
Yes	196	31.3	0.96	0.77–1.20	7.7	1.06	0.63–1.79
Improvement in at least one of the three stressors							
No	893	33.0	1		6.7	1	
Yes	587	31.4	0.95	0.82–1.11	8.7	1.29	0.90–1.85
Improvement in at least two of the three stressors							
No	1297	32.7	1		7.4	1	
Yes	169	36.3	1.11	0.89–1.37	10.8	1.47	0.92–2.36

CI: confidence interval; RR: risk ratio.

We performed various sensitivity tests with the whole sample (all age groups). We excluded workers who had moderate or severe mental distress in 2019 or 2020 from the propensity scores. We also included only workers who had high job demands, low job rewards, or low workplace social capital in 2019 in the propensity scores. We used the comparison group of workers who did not have any positive change in their psychosocial factors between 2019 and 2020 for the workers who had a significant decrease in two out of the three psychosocial factors during the same period. These tests did not alter the results.

## Discussion

The results of this quasi-experiment indicate that improving psychosocial stressors affects the risk for psychological distress differently among younger and older health and social care workers. The beneficial effect of psychosocial risk reduction was stronger among younger than older workers. Improving at least two of the three stressors – job demands, job rewards, and workplace social capital – were needed to reduce the risk of moderate or severe psychological distress among employees younger than 50 years. Among employees aged 50 years or older, improvement in job rewards reduced only the risk of mild psychological distress. The results indicate that future interventions should be tailored for workers in different ages.

The prevalence of moderate to severe psychological distress in 2021 was slightly higher than in the general population (9.5% vs. 8.7%, respectively) [18]. In the total sample, however, improving any of the stressors or a combination of two or three stressors resulted in an unsignificant change in psychological distress. The relatively low effect of improving psychosocial work stressors was a rather unexpected result, considering the effect of synergistically interacting work stressors on the risk of psychological distress, as shown by Nikunlaakso et al. [10]. In addition, following the rationale of Tsai and Venkataramani [11], as high job demands, low rewards, and low workplace social capital interact synergistically, we expected an intervention that affects two or three of the stressors to have a stronger effect on psychological distress. A possible explanation for the low effect of improving work stressors may be the outcome studied. For example, Halonen et al. [14] and Lallukka et al. [13], who found the disappearance of job strain and organisational injustice, respectively, to reduce the risk for insomnia symptoms. Similarly, Shiri et al. [15] found that using a participatory worktime scheduling software improved sleep problems and work ability, but not psychological distress. Conversely, Bourbonnais et al. [27] found an intervention improving several work stressors to reduce work-related burnout. Previous research and the findings from this study indicate that psychosocial work stressors may have a stronger impact on more direct work-related outcomes such as insomnia, burnout, and work ability, than psychological distress. Therefore, to improve workers’ mental health, factors other than work environment – presumably individual-level factors – should be addressed. Improving individual stress-coping and recovery skills could, for example, be beneficial.

To our knowledge, this is the first study that analyses the effect of improving accumulated work stressors on occupational health. It used a quasi-experimental design that followed a rigorous time order as the improvement of work stressors preceded psychological distress measurement. By using the design, we could balance the distribution of baseline covariates between quasi-intervention and quasi-control groups, mimicking a RCT study design. We did not collect any data on the methods used to reduce the stressors, though. Balancing the baseline characteristics of quasi-intervention and quasi-control groups ensured that the results were unaffected by the impact of COVID-19, as it was common in both groups.

The results of this study should be interpreted with caution due to some limitations. First, this study lacked some important baseline covariates, such as lifestyle variables, history of medical conditions, and body mass index. The covariates of this study included, however, self-perceived health, which estimates actual health well [28], and sleep problems. Second, the outcome measure was changed from baseline to follow-up. As both measures are validated to measure psychological distress, the change was unlikely to have affected the results. Third, the study lacked some psychosocial work stressors that may affect psychological distress, such as workplace bullying and low job control, which are known risk factors for depression [29]. Fourth, a further limitation is the substantial loss of participants in the survey sample due to the use of longitudinal data from three employee survey waves. Although propensity score weights were used to balance baseline covariates, this quasi-experiment is not equivalent to a RCT in this regard. Fifth, the study data were gathered using a survey that only had a shortened job demands scale, comprising two items which have, however, been found to associate with work disability [30]. Moreover, Fransson et al. [31] have found partial scales of job demands to show high to reasonable agreement with the complete scales. Sixth, the outcome used was self-reported and thus subject to reporting bias and temporal variation. We wanted, however, to study workers with increased psychological distress who still work actively and could thus benefit from interventions. Finally, the respondents of this study were social and healthcare workers from public sector organisations. The results cannot therefore be generalised to other working populations in which problems and needs might be different.

Despite the limitations, this study offers important implications. The findings of this study somewhat support the syndemics theory [11]: for employees aged under 50 years, improving two or three accumulated psychosocial stressors may reduce the risk for psychological distress. However, for older workers and in the total study population, improving psychosocial work stressors did not affect psychological distress. Although psychosocial work stressors might increase the risk for psychological distress [10], intervening in these stressors may have a limited effect when improving the situation, especially for older workers. This suggests, first, that employers should be proactive and prevent the accumulation of psychosocial work stressors in the first place. Second, organisational-level interventions that tackle psychosocial work stressors might reduce the risk for mental health problems, but the effect may vary. Individual-level interventions that tackle personal-level mental health challenges are also needed.

The findings of this study suggest several future study prospects. First, as both organisational-level and individual-level factors [32] seem to affect mental health, new workplace mental health interventions should include both organisational-level and individual-level elements. Organisational-level elements should aim to influence work environment and psychosocial stressors, and individual-level elements should aim to improve, for example, stress-coping skills. Second, as psychosocial work stressors impact workers in different ways, also other outcomes than mental health should be studied; for example, work ability and burnout. The studies are encouraged to apply a quasi-experimental design with a propensity score method. Third, this study was conducted during the COVID-19 pandemic, which may have affected the psychosocial stressors and psychological distress of workers, as well as the number of workers who experienced improvements in work stressors.

## Supplemental Material

sj-docx-1-sjp-10.1177_14034948241242160 – Supplemental material for The effect of improving psychosocial stressors on psychological distress: a quasi-experiment of Finnish health and social care workers Supplemental material, sj-docx-1-sjp-10.1177_14034948241242160 for The effect of improving psychosocial stressors on psychological distress: a quasi-experiment of Finnish health and social care workers by Risto Nikunlaakso, Rahman Shiri, Tuula Oksanen and Jaana Laitinen in Scandinavian Journal of Public Health
