# Supplementary material for: The effect of improving psychosocial stressors on psychological distress: a quasi-experiment of Finnish health and social care workers
Source: Scand J Public Health. 2024 Apr 3;53(3):276–83. doi: 10.1177/14034948241242160 (PMC12012273; doi:10.1177/14034948241242160)
Supplement: sj-docx-1-sjp-10.1177_14034948241242160 – Supplemental material for The effect of improving psychosocial stressors on psychological distress: a quasi-experiment of Finnish health and social care workers [file sj-docx-1-sjp-10.1177_14034948241242160.docx]

Appendix 1. Baseline characteristics of eligible population, respondents of the baseline survey, and final sample.

|  | **Eligible population**  **(N = 18 274)** | **Baseline respondents (N = 12 687)** | **Final sample**  **(N = 3 605)** |
| --- | --- | --- | --- |
| **Age (mean)** | 44.2 | 44.1 | 46.1 |
| **Gender** |  |  |  |
| Women | 86.7 | 88.0 | 88.0 |
| Men | 13.3 | 12.0 | 12.0 |
| **Occupation** |  |  |  |
| Physiotherapists and occupational therapists | 9.7 | 10.9 | 4.5 |
| Managers and administrative specialists | 3.3 | 3.9 | 5.4 |
| Kitchen workers | 0.2 | 0.1 | 0.4 |
| Laboratory technicians | 0.8 | 0.7 | 0.3 |
| Practical nurses | 31.6 | 29.0 | 26.2 |
| Physicians | 6.8 | 5.0 | 5.0 |
| Ward clerks | 2.6 | 2.8 | 2.7 |
| Psychologists, psychotherapists, and speech therapists | 1.3 | 1.3 | 1.1 |
| Radiological technologists | 0.8 | 0.7 | 0.6 |
| Nurses and midwives | 25.9 | 27.6 | 31.0 |
| Cleaners and nursing assistants | 2.8 | 2.2 | 3.2 |
| Social and other advisors | 7.5 | 8.4 | 11.1 |
| Social workers | 2.8 | 3.0 | 3.5 |
| Clerical workers | 2.3 | 2.6 | 2.6 |
| Head nurses | 1.0 | 1.2 | 1.7 |
| Other / miscellaneous | 0.6 | 0.6 | 0.6 |
